# Beware of Misdelivery: Multifaceted Role of Retromer Transport in Neurodegenerative Diseases

**DOI:** 10.3389/fnagi.2022.897688

**Published:** 2022-05-06

**Authors:** Shun Yoshida, Takafumi Hasegawa

**Affiliations:** ^1^Division of Neurology, Department of Neuroscience and Sensory Organs, Tohoku University Graduate School of Medicine, Sendai, Japan; ^2^Department of Neurology, National Hospital Organization Yonezawa Hospital, Yonezawa, Japan

**Keywords:** retromer, membrane trafficking, neurodegeneration, Alzheimer’s Disease, Parkinson’s disease

## Abstract

Retromer is a highly integrated multimeric protein complex that mediates retrograde cargo sorting from endosomal compartments. In concert with its accessory proteins, the retromer drives packaged cargoes to tubular and vesicular structures, thereby transferring them to the *trans*-Golgi network or to the plasma membrane. In addition to the endosomal trafficking, the retromer machinery participates in mitochondrial dynamics and autophagic processes and thus contributes to cellular homeostasis. The retromer components and their associated molecules are expressed in different types of cells including neurons and glial cells, and accumulating evidence from genetic and biochemical studies suggests that retromer dysfunction is profoundly involved in the pathogenesis of neurodegenerative diseases including Alzheimer’s Disease and Parkinson’s disease. Moreover, targeting retromer components could alleviate the neurodegenerative process, suggesting that the retromer complex may serve as a promising therapeutic target. In this review, we will provide the latest insight into the regulatory mechanisms of retromer and discuss how its dysfunction influences the pathological process leading to neurodegeneration.

## Introduction

Membrane trafficking is an evolutionarily conserved cellular process by which proteins and other macromolecules reach their destinations without crossing a membrane. Multiple lines of evidence have revealed that the defects in membrane trafficking are profoundly involved in the pathogenesis of neurodegenerative diseases (Hasegawa et al., 2017a). In particular, much interest has been focused on retromer because recent genetic and biological studies have underscored the significance of the retromer sorting machinery in the pathogenesis of Alzheimer’s Disease (AD) and Parkinson’s disease (PD) (Zhang et al., 2018). Retromer is considered a master regulator of retrograde cargo trafficking, e.g., transport from early endosomes (EEs) to the *trans*-Golgi network (TGN) and the plasma membrane (Seaman, 2021). On the other hand, retromer participates in the mitochondrial dynamics and the autophagic system, which are key processes in the maintenance of neuronal homeostasis (Cui et al., 2018). Moreover, pharmacological chaperones that stabilize retromer function successfully prevent neurodegeneration in cellular and animal models, suggesting that retromer is a promising target for disease-modifying therapy (Seaman, 2021). In this review, we will summarize the molecular basis of retromer function and discuss its pleiotropic roles in the causation and prevention of neurodegeneration.

## Retromer: A Master Regulator of Endosomal Sorting and Beyond

The term “retromer” was first used to describe an essential protein complex for the transport of vacuolar protein sorting 10p (Vps10p), a transmembrane receptor, from endosomes to the TGN in *Saccharomyces cerevisiae* (Seaman et al., 1998). Structurally, the retromer complex comprises five distinct proteins, namely Vps26p, Vps29p, Vps35p, Vps5p, and Vps17p.

**FIGURE 1:**
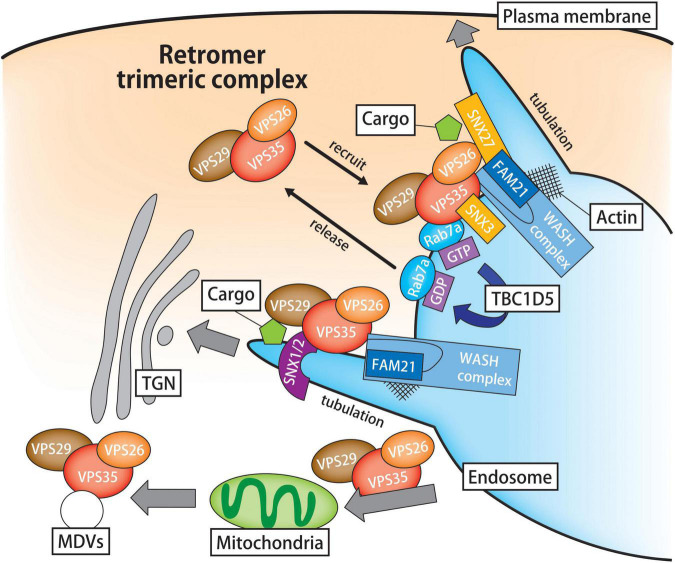
Schematic illustration of the retromer-mediated sorting pathway. Retromer is a hetero-trimeric protein complex composed of VPS26, VPS29, and VPS35. In cooperation with its associated proteins, the retromer sorting machine plays a primary role in the retrograde cargo trafficking from the endosomes to the TGN or plasma membrane. In the first step of retromer-mediated cargo transport, the retromer core is recruited to the endosomal surface under the control of Rab7a and SNX3. Because the endosomal recruitment of retromer complex largely depends on Rab7 activity, the inactivation of Rab7a by TBC1D5 promotes the release of retromer from endosomes. Lipid membrane deformation is crucial for intracellular trafficking and organelle remodeling. In the endosomal microdomain, the WASH complex modulates the process of endosomal tubulation through the activation of actin nucleation and polymerization. In addition to endosomal transport, retromer participates in the mitochondria dynamics through the cargo trafficking from the endosomes to the mitochondria and MDV-mediated trafficking. VPS, vacuolar protein sorting; TGN, *trans*-Golgi network; SNX, sorting nexin; TBC1D5, TBC1 domain family member 5; WASH, Wiskott-Aldrich Syndrome protein and scar homolog.

In mammals, retromer usually comprises a VPS26/VPS29/VPS35 heterotrimer complex because it lacks robust interaction with sorting nexin 1 (SNX1), a mammalian homolog of Vps5p (Seaman, 2021). In cooperation with the VPS26/VPS29/VPS35 trimeric structure, SNX orchestrates endosomal cargo sorting (Figure 1). Similarly, as their yeast counterpart Vps5p, SNX1 and SNX2 in mammals carry a Bim/Amphiphysin/Rvs (BAR) domain that drives membrane curvature and tubulation on the endosomal membrane (Carlton et al., 2005). Although the affinity of SNX1/2 for retromer is rather weak and transient, these SNX synergistically function in cargo retrieval from endosomes to the TGN (Bujny et al., 2007; Rojas et al., 2007). In addition, SNX5 and SNX6, which form heterodimers with SNX1/2, can directly interact with retromer cargoes such as cation-independent mannose-6-phosphate receptor (CI-MPR) (Wassmer et al., 2009; Simonetti et al., 2017; Yong et al., 2020). Likewise, SNX27 binds retromer subunit VPS26, and these proteins cooperatively drive the cargo transport from endosomes to the plasma membrane (Steinberg et al., 2013; Gallon et al., 2014).

In the first step of retromer-dependent cargo transport, the retromer core is recruited to the endosomal surface under the control of Rab7a and SNX3 (Rojas et al., 2008; Seaman et al., 2009). Interestingly, the inactivation of Rab7a by TBC1 domain family member 5 (TBC1D5), a Rab7a GTPase-activating protein (GAP), promotes the release of retromer from endosomes (Seaman et al., 2009; Ye et al., 2020). Meanwhile, SNX3 binds to the endosomal membrane, thereby initiating retromer-mediated retrograde transport irrespective of SNX1/2 and SNX5/6 (Strochlic et al., 2007; Harterink et al., 2011). In the endosomal microdomain, the Wiskott-Aldrich syndrome protein and scar homolog (WASH) complex modulates the process of endosomal tubulation (Linardopoulou et al., 2007; Gomez and Billadeau, 2009) (Figure 1). The WASH molecular machinery is a macromolecular protein complex composed of WASH1, FAM21, strumpellin- and WASH-interacting protein, strumpellin and coiled-coil domain-containing protein 53. VPS35 interacts with the unstructured C-terminal tail of FAM21 and recruits FAM21 to endosomes, whereas the FAM21-WASH interaction occurs through its N-terminus, thereby regulating actin polymerization (Harbour et al., 2012; Hao et al., 2013). Additionally, FAM21 interacts with SNX27, directing SNX27-retromer cargoes to the plasma membrane (Temkin et al., 2011).

Apart from the endosomal cargo sorting, the retromer is involved in mitochondrial dynamics and the autophagic system (Figure 1). An unbiased molecular screening revealed that both VPS35 and VPS26 bind the mitochondrial-anchored protein ligase (MAPL) (Braschi et al., 2010). The recruitment of VPS35 to mitochondria regulates the transport of MAPL to peroxisomes via mitochondrial-derived vesicles (MDVs). Moreover, VPS35 participates in the recycling of dynamin-like protein 1 (DLP1), a mitochondrial fission protein (Wang W. et al., 2016). In that sense, retromer may control mitochondrial dynamics through cargo-protein trafficking. Another line of evidence suggested a regulatory role for retromer in autophagy lysosomal pathway. Actually, proteomics analysis of autophagosome composition in MCF7 cells identified VPS35 as an autophagosome-associated protein (Dengjel et al., 2012). When retromer is depleted, Atg9 aberrantly remains in EEs and interferes with subsequent autophagosome formation (Ravussin et al., 2021). In addition, retromer participates in mitophagy by regulating Rab7 activity with TBC1D5 (Jimenez-Orgaz et al., 2018). Cumulatively, these findings provide a scientific basis for the fundamental role of retromer in the maintenance of cellular homeostasis and stress tolerance.

## Alzheimer’s Disease

### Genetic and Pathological Evidence Linking Retromer and Alzheimer’s Disease

Alzheimer’s Disease (AD) is the most common cause of progressive dementia among older populations. The histopathological signature of AD is the deposit of extracellular senile plaques and intracellular neurofibrillary tangles, which are composed mainly of aggregated amyloid-β (Aβ) and phosphorylated tau, respectively (DeTure and Dickson, 2019). The synergistic neurotoxicity of these two proteins in AD has been extensively studied, and growing evidence suggests that the retromer sorting pathway exerts a substantial impact on the generation of AD pathology through Aβ production and tau accumulation (Zhang et al., 2018). Microarray analysis using the entorhinal cortex and the dentate gyrus from the autopsied brain tissue of patients with AD demonstrated that the expression of the retromer subunits VPS35 and VPS26 is markedly reduced at both the mRNA and protein levels. This finding is further corroborated by experiments in a cultured cellular model revealing that VPS35 silencing leads to a significant increase of endogenous Aβ level (Small et al., 2005). Like AD, the expression level of VPS35 is significantly decreased in the brains of patients with distinct primary tauopathies such as progressive supranuclear palsy (PSP) and Pick’s disease, and downregulation of VPS35 results in the exacerbation of motor and learning impairments and accumulation of pathological tau in a relevant mouse model (Vagnozzi et al., 2019). Moreover, a gene-association study between AD and single nucleotide polymorphisms (SNPs) in 15 retromer-related genes revealed a positive association for several retromer-associated genes (e.g., *SNX3*, *RAB7A*, *KIAA1033*, and *SNX1*) (Vardarajan et al., 2012). Furthermore, copy number variation analysis and whole exome sequencing in sporadic early-onset AD identified a *de novo* deleterious variant (L625P) in *VPS35* in a French cohort (Rovelet-Lecrux et al., 2015). The pathogenic role of retromer in AD is also supported by studies using different animal models. Human amyloid precursor protein (APP) transgenic (Tg) mice (Tg2576 and J20) exhibit a progressive decrease in the expression levels of VPS35, VPS26, and CI-MPR (Chu and Praticò, 2017; Tammineni et al., 2017). In *Macaca fascicularis*, an age-dependent decline in the endosomal sorting machinery including the retromer is closely related to the intracellular accumulation of APP and Aβ (Kimura et al., 2009, 2016). Lifestyle-related diseases, such as hypertension and diabetes, are known as major risk factors for AD, and interestingly, a mouse model of type 2 diabetes revealed hippocampus-specific retromer deficiency similarly as observed in an APP Tg mouse model (Morabito et al., 2014).

### Roles of Retromer in the Trafficking and Metabolism of Alzheimer’s Disease-Related Proteins

A plethora of evidence suggests that the endosomal sorting machinery including the retromer has a great impact on the biogenesis and transport of Aβ peptides in healthy and diseased brains (Willén et al., 2017; Kimura and Yanagisawa, 2018). Cellular and animal model studies demonstrated that retromer deficiency facilitates the buildup of toxic Aβ oligomers in the endosomal compartments, resulting in abnormal endosomal enlargement and subsequent neuronal cell death (Muhammad et al., 2008; Wen et al., 2011; Bhalla et al., 2012; Ansell-Schultz et al., 2018). In the amyloidogenic pathway, Aβ synthesis is initiated through the proteolytic cleavage of APP by β-secretase [β-APP-cleaving enzyme-1 (BACE1)] on the plasma membrane, TGN, and EEs, followed by the transport to the multivesicular bodies (MVBs) (Rajendran et al., 2006; Burgos et al., 2010; Willén et al., 2017). BACE1 produces the N-terminal fragment of APP called soluble peptide APPβ, and the C-terminal fragment of APP named β-CTF (also known as C99). Subsequently, the Aβ peptide is generated as a fragment, in which β-CTF is cleaved by γ-secretase within endosomes. Importantly, the retromer complex contributes to the retrograde transport of APP, BACE1, γ-secretase, and related proteins from the endosomes, and thus, its malfunction causes the aberrant endosomal retention of these molecules, leading to the overproduction of Aβ (Wen et al., 2011; Bhalla et al., 2012; Choy et al., 2012; Cuartero et al., 2012; Kanatsu et al., 2018).

**FIGURE 2:**
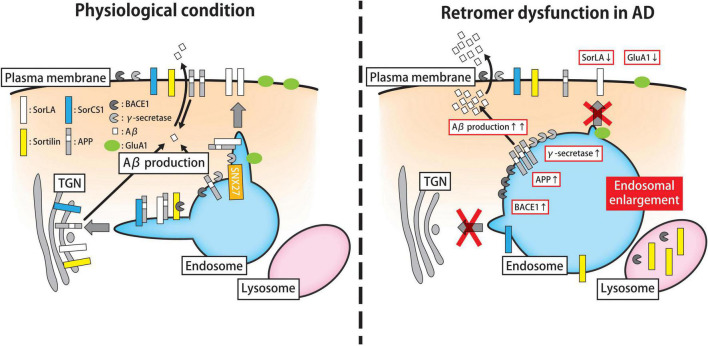
Roles of the retromer machinery in the trafficking and metabolism of AD-related proteins. Endosomal trafficking plays a key role in the processing of APP and the biogenesis of Aβ peptides. Under physiological conditions (*left panel*), the Aβ peptide is synthesized through the proteolytic cleavage of APP by BACE1 (also known as β-secretase) and γ-secretase on the plasma membrane, early endosomes, and TGN. Retromer complex contributes to the retrograde transport of APP, BACE1, γ-secretase, and related proteins (e.g., SorLA and SorCS1) from endosomes. The SorLA-mediated transport of APP to the plasma membrane requires the support of SNX27. In addition to the endosomal pathway, the retromer might participate in surface recycling of the AMPAR subunit GluA1, thereby modulating synaptic plasticity. In AD brains, in which retromer function is compromised (*right panel*), APP, BACE1, γ-secretase, and associated proteins accumulate in early endosomes, thereby increasing Aβ production with hypertrophic changes in endosomal compartments. Besides the amyloidogenic pathway, retromer malfunction perturbs the cell surface recycling of GluA1, which may influence on synaptic plasticity. APP, amyloid precursor protein; Aβ, amyloid-β; BACE1, β-APP-cleaving enzyme-1; TGN, *trans*-Golgi network; SNX sorting nexin; AMPAR, α-amino-3-hydroxy-5-methyl-4-isoxazole propionate receptor; AD, Alzheimer’s Disease.

The retrograde trafficking of APP is mediated by the Vps10 domain-containing proteins SorLA and sortilin-related Vps10 domain-containing receptor 1 (SorCS1) through their interactions with the retromer complex (Pallesen and Vaegter, 2012) (Figure 2). Intriguingly, the *SORL1* gene, a gene encoding SorLA which is abundantly expressed in the central nervous system, is associated with both late- and early-onset forms of AD (Rogaeva et al., 2007; Pottier et al., 2012). In addition, the protein expression of SorLA is significantly lower in brain tissue and cerebrospinal fluid (CSF) from patients with sporadic AD compared to controls, (Scherzer et al., 2004; Ma et al., 2009). Intriguingly the reduced expression of SorLA in the brains begins even in the prodromal phase of AD, and low SorLA expression is correlated with cognitive function (Sager et al., 2007). In agreement with these findings, overexpression of SorLA decreased the expression level of APP and Aβ in cellular and mouse models, whereas loss of SorLA increased the Aβ load (Andersen et al., 2005; Offe et al., 2006; Schmidt et al., 2007; Dodson et al., 2008). Mechanistically, SorLA co-localizes with APP in EEs, and transports APP to the TGN in association with the retromer complex (Andersen et al., 2005; Fjorback et al., 2012). Collectively, these results suggest that the lack of interaction between APP and SorLA perturbs the retrograde trafficking of APP and SorLA from EEs to the TGN or plasma membrane, leading to aberrant endosomal retention of APP and Aβ in the AD brain.

Notably, the SorLA-mediated retrieval of APP to the plasma membrane is regulated by SNX27; thus, the depletion of SNX27 leads to the accumulation of SorLA and APP in EEs, thereby promoting Aβ production (Huang et al., 2016). Indeed, the coding variants of *SORL1* identified in the familial and sporadic forms of AD bind APP less well, and HEK293 cells overexpressing mutant *SORL1* displayed increased Aβ secretion in culture medium (Vardarajan et al., 2015). Similarly, SorLA-deficient human induced pluripotent stem cell (iPSC)-derived neurons specifically exhibit the abnormal enlargement of EEs with APP accumulation, which mimics affected neurons in AD brains (Cataldo et al., 2000; Knupp et al., 2020). Moreover, genetic cohort studies demonstrated that variants in *SORCS1*, a gene encoding another Vps10 family protein, are significantly associated with AD as well as type 1 and type 2 diabetes mellitus (Goodarzi et al., 2007; Paterson et al., 2010; Reitz et al., 2011). Similarly as SorLA protein, overexpression of SORCS1 in HEK293 cells transfected with mutant APP reduces Aβ secretion into the culture medium. Conversely, SORCS1 silencing significantly increases Aβ secretion together with the decline of VPS35 level in the mice brain (Lane et al., 2010; Reitz et al., 2011). Regarding the mode of action, it is likely that SORCS1 does not directly modulate the endocytic uptake of APP, but rather, it regulates the exit of APP and/or CTFs out of EEs, resulting in increased APP translocation to the TGN as well as decreased Aβ secretion (Lane et al., 2010, 2013).

The scission of APP by BACE1 is putatively the rate-limiting step in Aβ synthesis. As a type 1 transmembrane aspartic protease, BACE1 activity is highest in acidic compartments including the endosomal compartments and TGN, making it plausible that the regulation of the post-Golgi transport of BACE1 plays an important role in the processing of APP and Aβ genesis (Sun and Zhang, 2017). In neuronal cells, BACE1 is transported from EEs to the TGN through retromer-mediated transport, and thus, the loss of retromer function promotes the retention of BACE1 in endosomes, resulting in increased binding of APP to BACE1 and consequently, Aβ production (Wen et al., 2011; Cuartero et al., 2012) (Figure 2). During this process, sortilin, a Vps10 domain-containing protein, cooperatively regulates the trafficking of BACE1. Specifically, sortilin on the cell surface is taken up by cells via adaptor protein 1-dependent endocytosis, which is followed by transport to the TGN with BACE1 (Canuel et al., 2008; Finan et al., 2011). The expression level of sortilin is correlated with Aβ production and is markedly elevated in the brain tissue of patients with AD and retromer-deficient mice (Kim et al., 2010; Finan et al., 2011). Supporting this result, the N-terminal fragments of two BACE1 substrates, namely APP-like 1 and close homolog of L1, are substantially increased in the CSF of forebrain-specific Vps35 KO mice and patients in the prodromal stage of AD (Simoes et al., 2020). Altogether, these findings strongly suggest that the subcellular trafficking of BACE1 and the amyloidogenic APP processing pathway largely depend on the retromer function.

In addition to the amyloidogenic pathway, the retromer machinery is likely to modulate the subcellular trafficking of cargo molecules related to AD pathogenesis. One example is triggering receptor expressed on myeloid cells 2 (TREM2), a risk gene for AD and an important regulator of microglial functions (Guerreiro et al., 2013; Jonsson et al., 2013; Lee et al., 2018). TREM2 is a transmembrane receptor of the immunoglobulin superfamily that is mainly expressed in monocytes, macrophages, dendritic cells, and microglia, and it undergoes shutting between the plasma membrane and endosomal compartments in association with retromer (Yin et al., 2016). Particularly, the loss of retromer components perturbs plasma membrane-resident TREM2 but increases its lysosomal translocation for degradation, which impairs the microglial activation and phagocytic clearance of Aβ (Lucin et al., 2013; Yin et al., 2016). Consistent with this finding, R47H TREM2, an AD-associated mutant, disrupts the binding to VPS35, and it is destined for lysosomal degradation (Yin et al., 2016). The importance of retromer function in the microglial clearance of Aβ is further supported by a recent study showing that microglia-specific Vps35 conditional KO 5XFAD mice showed impaired microglial uptake of Aβ and disease-associated microglia development in the brains, resulting in the exacerbation of Aβ-related pathology and cognitive decline (Ren et al., 2022).

Other evidence demonstrated the putative role of retromer in the alteration of synaptic plasticity in AD (Figure 2). In hippocampal neurons, VPS35 deficiency impairs the surface recycling of α-amino-3-hydroxy-5-methyl-4-isoxazole propionate receptor (AMPAR) subunit GluA1 during long-term potentiation (LTP), resulting in dendritic spine deficit (Tian et al., 2015; Temkin et al., 2017). In addition, Vps26B, a brain-enriched paralog of Vps26 in mammals, potentiates the activity-dependent retrograde trafficking of GluA1 during LTP (Bugarcic et al., 2011; Simoes et al., 2021). It is interesting that silencing of Vps26B, but not Vps26A, in mice significantly decreases SorLA levels in the plasma membrane, which is accompanied by increased Aβ and tau accumulation in brain tissue and CSF (Simoes et al., 2021).

### Considerations for Retromer as a Therapeutic Target in Alzheimer’s Disease

Given the multifaceted roles of retromer in the subcellular trafficking of AD-related proteins, one can imagine that the genetic engineering or pharmacological stabilization of retromer components may have a potentially beneficial effect on the neurodegenerative process of AD. For example, intracerebral AAV-mediated gene transfer of VPS35 in triple Tg (3xTg) mice {i.e., a human mutant presenilin 1 [M146V] knockin (KI), mutant APP [KM670/671NL] and tau [P301L] transgene} ameliorates cognitive dysfunction, which is associated with significant decreases in Aβ deposition and phosphorylated tau levels (Li et al., 2020a). Moreover, overexpression of VPS35 in cultured cellular models increases the expression of cathepsin D (CTSD), a lysosomal aspartic protease, thereby promoting the autophagic clearance of pathological tau aggregates (Vagnozzi et al., 2019). In addition to retromer gene transduction, retromer stabilization by the chemical chaperones R33 and R55 can mitigate AD-related pathology. Specifically, both R33 and R55 stabilize the retromer complex by binding the interface between VPS35 and VPS29, thereby preventing their degradation (Mecozzi et al., 2014). In the aforementioned 3xTg mice, R33 successfully prevented memory deficit along with reducing the intracerebral Aβ burden and phosphorylated tau levels (Li et al., 2020b). Likewise, in human iPSC-derived neurons from patients with AD, both R33 and R55 reduced tau phosphorylation in an APP-independent manner (Young et al., 2018). Finally, a recent cellular and animal model study demonstrated that the administration of R33 ameliorated the retention of APP in EE and increased the level of phosphorylated tau under high glucose condition (Chae et al., 2022). Taken together, these results open up a new therapeutic avenue for targeting retromer in AD. Future studies are required to further evaluate the efficacy of retromer-modulating drugs in different types of cellular and animal models.

## Parkinson’s Disease

### Genetic Basis Linking Retromer and Parkinson’s Disease

Parkinson’s disease (PD), the second most common neurodegenerative disease, is clinically characterized by a progressive movement disability and a variety of non-motor symptoms. The neuropathological hallmarks of PD are the preferential loss of dopaminergic neurons in the substantia nigra *pars compacta* (SNpc) and the appearance of cytoplasmic inclusions called Lewy bodies (LBs), which are mainly composed of hyperphosphorylated, aggregated α-synuclein (α-syn) (Baba et al., 1998).

After the discovery of the missense mutations in the *VPS35* gene in a late-onset, dominantly inherited familial form of PD (PARK17), the retromer function in the pathogenesis of PD has been highlighted (Vilariño-Güell et al., 2011; Zimprich et al., 2011). The exogenous induction of PD-related *leucine-rich repeat kinase 2* (*LRRK2*, PARK8) and *Rab7L1* also impairs the retromer-mediated transport of MPR with abnormal lysosomal swelling. Somewhat surprisingly, the expression of wild-type (WT) VPS35 (^WT^VPS35) can rescue the phenotypes induced by *LRRK2* or *RAB7L1* variants both *in vitro* and *in vivo*, suggesting that these three genes might operate in a common cellular pathway (MacLeod et al., 2013). Moreover, in the brain tissue from *LRRK2* mutation carriers, the insoluble form of VPS35 is prominently increased probably because of retromer or lysosomal dysfunction (Zhao et al., 2018). Although the mechanisms by which VPS35 and LRRK2 synergistically participate in the pathogenesis of PD remains unclear, several possibilities have been postulated. The pathogenic D620N VPS35 (^D620N^VPS35) mutant enhanced LRRK2-mediated Rab10 phosphorylation in cellular and mouse models. Conversely, an *in vivo* study using a fly model revealed that *Drosophila vps35* (*dvps35*) and *LRRK2* cooperatively modulate synaptic vesicle endocytosis through the endosomal pathway (Inoshita et al., 2017; Mir et al., 2018). Several lines of evidence also suggest a molecular interaction between *VPS35* and *PARKIN* (PARK2), the most common cause of autosomal recessive young-onset parkinsonism. In a *Drosophila* model, *vps35* genetically interacted with *PARKIN* but not with *PINK1* (*PTEN-induced putative kinase 1*), and notably, *vps35* overexpression rescued several parkin-mutant phenotypes (Malik et al., 2015). As an E3 ubiquitin ligase, parkin directly interacts with VPS35 through its RING1 domain, thereby modulating retromer function through VPS35 ubiquitination (Williams et al., 2018). Additionally, as a vesicle-associated protein, α-syn can influence retromer-mediated sorting by interfering with the interaction between SNX3 and PI(3)P (Patel et al., 2018; Kobayashi et al., 2019). Another interesting finding was that the loss of *iPLA2-VIA*, a *Drosophila* homolog of *PLA2G6* (PARK14), inhibits the retromer-mediated transport of sphingolipids from endosomes to the TGN, resulting in the lysosomal dysfunction due to ceramide overload in the lysosomes (Lin et al., 2018). Intriguingly, similar results were observed upon loss of *vps26* or *vps35* or overexpression of α-syn in this fly model, indicating that these defects might be common in the pathogenesis of PD. In addition to genetic models mimicking familial forms of PD, VPS35 overexpression may have a protective effect on toxin-induced models such as rotenone-induced *Drosophila* PD model (Linhart et al., 2014; Dhungel et al., 2015; Williams et al., 2018). Taken together, these findings indicate that the retromer sorting machinery may configure a common biological pathway involved in PD.

### Molecular and Cellular Mechanisms Underlying Familial Parkinson’s Disease With Retromer-Related Gene Mutations

Although the molecular mechanism underlying neuronal loss in PD remains unclear, the critical roles of endosomes and their associated trafficking process in the pathophysiology of PD have emerged (Hasegawa et al., 2011, 2017a,b; Konno et al., 2012; Sugeno et al., 2014; Yoshida and Hasegawa, 2022). In particular, the discovery of VPS35 as a responsible gene for PARK17 has attracted great attention because this finding revealed a causal relationship between the retromer machinery and PD (Vilariño-Güell et al., 2011; Zimprich et al., 2011). Among the VPS35 mutations so far identified, the D620N missense mutation in the C-terminus of VPS35 has been consistently reported in unrelated PD families from different ethnicities (Sassone et al., 2021). Based on these results, genetically engineered animal models harboring ^D620N^VPS35 have been created, and they have variable phenotypes. In a viral-mediated gene transfer rat model, the expression of human ^D620N^VPS35 in the SNpc resulted in prominent dopaminergic neuron loss with axonal pathology, whereas ^D620N^VPS35 Tg aged mice generated via Rosa26-based transgenesis did not exhibit apparent motor impairment or neurodegeneration (Tsika et al., 2014; Vanan et al., 2020). On the contrary, Tg flies expressing human ^D620N^VPS35 or ^P316S^VPS35 displayed a detrimental phenotype including dopaminergic neuron loss, locomotor dysfunction, a shortened lifespan, and susceptibility toward PD-linked environmental toxins (Wang et al., 2014). There are several conflicting research findings about ^D620N^VPS35 KI mice; however, some of them exhibit levodopa-responsive motor impairment with dopaminergic neuron loss in the SNpc (Chen et al., 2019; Chiu et al., 2020; Niu et al., 2021). Notably, ^D620N^VPS35 KI mice display phosphorylated tau accumulation and tangle-like pathology instead of LB pathology (Ishizu et al., 2016; Chen et al., 2019; Chiu et al., 2020), which may have mimicked the autopsy findings in the Japanese PARK17 patient carrying *VPS35* mutation displaying “pure nigral” degeneration without LB pathology in the brain (Bono et al., 2020).

**FIGURE 3:**
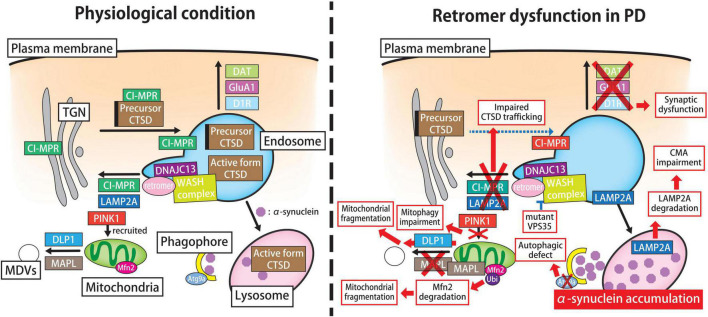
Retromer-mediated pathogenic pathway in PD. In physiological states (*left panel*), the retromer participates in the activation and CI-MPR-mediated sorting of CTSD, a major lysosomal hydrolase for α-syn degradation. The clearance of neurotoxic α-syn species largely depends on the autophagy-lysosomal pathway, especially macroautophagy and CMA. Retromer modulates the retrograde transport of LAMP2A, a receptor for CMA, from endosomes to the TGN, thereby preventing its degradation in lysosomes. In addition, retromer regulates the surface recycling of GluA1, D1R and DAT and thus influences synaptic function. Furthermore, retromer might participate in the autophagic machinery and mitochondrial dynamics, which are key processes in the maintenance of neuronal homeostasis. In the PD brain (*right panel*), retromer function is disturbed by aging, environmental toxin exposure, or genetic alterations. The familial PD-linked mutant VPS35 disrupts the molecular interaction between VPS35 and the WASH complex, which hampers retromer-mediated cargo sorting to the TGN. The perturbation of endosome-TGN trafficking hampers CTSD trafficking, thereby leading to abnormal α-syn accumulation in lysosomes. Likewise, retromer failure impairs the endosome-to-TGN retrieval of LAMP2A and accelerates its degradation in lysosomes, which compromises the CMA-mediated degradation of cytotoxic α-syn. Moreover, mutant VPS35 causes autophagic failure, which is partly explained by the mislocalization of autophagy protein ATG9A. Additionally, retromer failure increases the expression level of GluA1 and D1R on the plasma membrane, and thus induces synaptic dysfunction. In addition, mutant VPS35 has detrimental effects on mitochondrial fission-fusion dynamics and PINK1-PARKIN-mediated mitophagy, which results in mitochondrial dysfunction and fragmentation. The PD-linked DNAJC13 mutation influences on the trafficking of multiple cargoes possibly due to the alteration of the membrane dynamics via retromer-related WASH complex and SNX1. CI-MPR, cation-independent mannose-6-phosphate receptor; CTSD, cathepsin D; CMA, chaperone-mediated autophagy; LAMP2A, lysosomal-associated membrane protein 2A; TGN, *trans*-Golgi network; D1R, dopamine D1 receptor; DAT, dopamine transporter; PD, Parkinson’s disease; VPS, vacuolar protein sorting; WASH, Wiskott-Aldrich Syndrome protein and scar homolog; ATG, autophagy-related; PINK1, PTEN-induced putative kinase 1; SNX sorting nexin; DNAJC13, DnaJ heat shock protein family (Hsp40) member C13.

The mechanisms by which mutant VPS35 induces the PD-related pathology remain uncertain; however, several possibilities have been proposed: (i) toxic α-syn accumulation attributable to lysosomal dysfunction, (ii) synaptic dysfunction, and (iii) impaired mitochondrial dynamics and mitophagy (Figure 3). Retromer plays a key role in the lysosomal sorting of CTSD, a major lysosomal hydrolase in α-syn degradation (Sevlever et al., 2008). Namely, upon arrival in the Golgi apparatus, newly synthesized lysosomal enzymes including CTSD are modified with mannose 6-phosphate residues, which are recognized by CI-MPR in the TGN. CTSD is translocated to endosomes and released for further sorting to lysosomes. Retromer retrieves the unoccupied CI-MPR from endosomes to the TGN, where they participate in further cycles of CTSD sorting. Hence, retromer malfunction decreases the levels of the active form of CTSD in lysosomes and thus leads to abnormal α-syn accumulation (Follett et al., 2014; Miura et al., 2014). Through comparative stable isotope labeling by amino acids in cell culture (SILAC)-based analysis, the major defect of ^D620N^VPS35 is attributed to its insufficient interaction with the actin-nucleating WASH complex, which results in perturbation of endosome-to-TGN trafficking (McGough et al., 2014). Likewise, exogenous expression of ^D620N^VPS35 in HeLa cells can rescue lysosomal proteolytic defect and altered autophagic flux caused by the silencing of endogenous VPS35; however, this mutant fails to support the retrieval of CI-MPR from endosomes to the TGN (Cui et al., 2021). Similarly as ^D620N^VPS35, ^R524W^VPS35 and ^A320V^VPS35 can also interfere with retrograde cargo sorting in the endosome-to-TGN pathway (Follett et al., 2016; Wu et al., 2020).

In PD and other synucleinopathies, one of the major concerns is the mode of α-syn clearance. Although some researchers have emphasized the importance of the ubiquitin-proteasome system for α-syn degradation, numerous studies have suggested that its degradation largely depends on the autophagy lysosomal pathway, especially macroautophagy and chaperone-mediated autophagy (CMA) (Cuervo et al., 2004; Oshima et al., 2016). Because retromer function is closely involved in the maintenance of autophagy-mediated proteostasis (Figure 3), it is easy to assume that retromer malfunction could influence the clearance of toxic α-syn and subsequent neurodegeneration. Indeed, PD-linked ^D620N^VPS35 impairs WASH complex recruitment to the endosomes and thus causes autophagic failure, which is partly explained by the mislocalization of the autophagy protein ATG9A (Zavodszky et al., 2014). Furthermore, dopaminergic neurons expressing ^D620N^VPS35 and neurons in ^D620N^VPS35 KI mice exhibit impaired endosome-to-Golgi retrieval of LAMP2A, thereby accelerating LAMP2A degradation in lysosomes (Tang et al., 2015a; Niu et al., 2021). Collectively, these results suggest that retromer malfunction impairs the cellular clearance of α-syn via the autophagy-lysosome pathway, thereby accelerating neurodegeneration possibly due to the accumulation of toxic, misfolded α-syn species.

Growing evidence suggests that retromer can influence mammalian nervous system development and synaptic neurotransmission in healthy and diseased brains (Brodin and Shupliakov, 2018). Although limited evidence is available, several studies claim that the disorder in retromer function by PD-related VPS35 mutations may affect synaptic function (Figure 3). In mouse primary cortical neurons, the presence of ^D620N^VPS35 was less frequently present in dendritic spines than ^WT^VPS35, and ^D620N^VPS35 tended to form clusters with FAM21 in EEs (Munsie et al., 2015; Kadgien et al., 2021). In the synaptic nerve terminal, VPS35 participates in the cell surface recycling of GluA1, dopamine D1 receptor (D1R), and dopamine transporter (DAT), and thus, ^D620N^VPS35 might increase the surface expression of these receptors, thereby producing chronic stress in neuronal circuits (Munsie et al., 2015; Wang C. et al., 2016; Wu et al., 2017; Kadgien et al., 2021).

Mitochondria have long been recognized as a key component in the pathogenesis of PD (Hasegawa et al., 2006; Bose and Beal, 2016). Another interesting idea is that the VPS35 pathogenic mutation may have a detrimental effect on mitochondrial fission-fusion dynamics and mitophagy (Figure 3). Indeed, aberrant mitochondrial fragmentation and impaired mitophagy have been observed in fibroblasts from patients bearing ^D620N^VPS35 (Wang W. et al., 2016; Hanss et al., 2021). The underlying mechanism of mitochondrial fragmentation induced by VPS35 deficiency is supposed to be aberrant trafficking of MAPL (also known as mitochondrial E3 ubiquitin ligase-1) and dynamin-like protein 1 (DLP1). More specifically, ^D620N^VPS35 impairs the trafficking of MAPL from mitochondria to MDVs, and the overloaded MAPL in mitochondria ubiquitinates mitofusin-2 (MFN2), thereby promoting mitochondrial fragmentation (Tang et al., 2015b). Alternatively, ^D620N^VPS35 enhances the VPS35-DLP1 interaction and increases the turnover of the DLP1 complex in mitochondria, which induces neurodegeneration by increasing the rate of mitochondrial fission (Wang W. et al., 2016). It is widely accepted that both PINK1 and PARKIN participate in the quality control pathway to sense damaged mitochondria and target them for degradation through mitophagy (Tanaka, 2020). A recent study using SH-SY5Y cells carrying the ^D620N^VPS35 demonstrated that mutant VPS35 impairs the PINK1-PARKIN-mediated mitophagy through impaired PINK1 recruitment to mitochondria, suggesting a converging pathophysiological cascade among VPS35, PINK1, and PARKIN in PD (Ma et al., 2021).

Another important player in the endosomal cargo sorting is a DNAJC13 [DnaJ heat shock protein family (Hsp40) member C13], which associates with SNX1 and has been linked to an autosomal-dominant, late-onset familial form of PD (PARK21) (McGough and Cullen, 2013; Freeman et al., 2014; Vilariño-Güell et al., 2014; Gustavsson et al., 2015). The neuropathological feature in *DNAJC13* N855S (^N855S^DNAJC13) mutation carriers is the presence of the brainstem or transitional type of LB pathology (Appel-Cresswell et al., 2014; Vilariño-Güell et al., 2014). DNAJC13 is a human homolog of receptor-mediated endocytosis 8 (RME-8) in nematodes and is ubiquitously expressed including the nervous system (Fujibayashi et al., 2008). Structurally, it includes four conserved IWN repeats, which are characterized by seven invariant residues, including isoleucine, tryptophan, and asparagine, and a Hsc70-binding J-domain (Hasegawa et al., 2017b). In a fly model, ^N855S^DNAJC13 exacerbated α-syn-mediated motor dysfunction, a rough eye phenotype, and the loss of dopaminergic neurons, which recapitulates the clinicopathological features of PD (Yoshida et al., 2018). In concert with SNX1 and FAM21, DNAJC13 is recruited to EEs, where it participates in multidirectional endosomal sorting including the retrieval of CI-MPR (Hasegawa et al., 2017b). It remains unclear whether the PD-linked DNJAC13 mutant could directly impede retromer function; however, the expression of ^N855S^DNAJC13 in cultured cells alters the membrane dynamics of retromer-related SNX1 and influences the trafficking of multiple cargoes, e.g., the transport of epidermal growth factor receptor (EGFR) to the lysosomes, the recycling of transferrin receptor (TfR) to the cell surface, notch receptor recycling, and the transport of ATG9A to the phagophores (Gomez-Lamarca et al., 2015; Yoshida et al., 2018; Follett et al., 2019; Besemer et al., 2021). Further work is required to precisely identify the pathophysiological role of DNAJC13 in the neurodegenerative process leading to PD.

### Retromer’s Roles in Other Neurodegenerative Diseases

Although there is only limited evidence available at present, the retromer sorting system may also contribute to the etiopathogenesis of less common neurodegenerative diseases such as amyotrophic lateral sclerosis (ALS) and Huntington’s disease (HD). Amyotrophic lateral sclerosis is a fatal neurodegenerative disease clinically characterized by the selective loss of both upper and lower motor neurons. Although most ALS cases are sporadic, approximately 10% of cases are familial (FALS) and predominantly associated with Mendelian-inherited mutations in genes including *Cu/Zn superoxide dismutase* (*SOD1*) and *C9ORF72*. Notably, the iPSC-derived motor neurons from patients with FALS carrying *C9ORF72* hexanucleotide repeat expansion or G93A SOD1 mutation exhibit retromer deficiency, and retromer stabilization by chemical chaperone attenuated the locomotive activity and motor neuron loss in G93A SOD1 Tg mice (Aoki et al., 2017; Muzio et al., 2020). Likewise, the neuron-specific deletion of VPS35 results in the selective loss of ventral horn motor neurons with the formation of p62-positive inclusions in the spinal cord, mimicking the neuropathological features of sporadic ALS (Sargent et al., 2021).

Huntington’s disease (HD) is a progressive, dominantly inherited neurodegenerative disorder clinically manifesting as involuntary movement and cognitive and psychiatric impairment. The cardinal genetic defect in HD is the abnormally elongated polyglutamine repeat expansion in the *huntingtin* (*HTT*), and striatal medium spiny neurons (MSNs) are known as the most vulnerable cells in HD. In MSNs, SorCS2 interacts with VPS35, thereby regulating the surface trafficking of the NR2A subunit of N-methyl-D-aspartate (NMDA) receptor. Intriguingly, SorCS2 selectively interacts with mutant huntingtin but not WT huntingtin, and it is mislocalized to perinuclear clusters in the striatal neurons of patients with HD and model mice, indicating that retromer affects the pathogenesis of HD by modulating SorCS2-mediated NR2A trafficking in MSNs (Ma et al., 2017).

## Concluding Remarks and Future Prospectives

In this review, we summarized the functional roles of the retromer as an endosomal trafficking regulator and its implication in the pathogenesis of neurodegenerative disorders, including AD, PD, ALS, and HD. In particular, after the discovery of missense mutations in VPS35, a core component of retromer, in autosomal dominant forms of PD, the role of retromer-mediated endosomal sorting came into the limelight in the etiopathogenesis of PD. However, several questions remain to be clarified (e.g., why neurons are selectively vulnerable to the retromer dysfunction; why do mutations in the same retromer-associated gene result in multiple phenotypes and different disorders; are disease-associated protein aggregates an indicator of retromer malfunction, a driver of retromer impairment, or both). Currently, most researchers postulate that defects in the retromer machinery affect the proteostasis and cellular burden of cytotoxic proteins including α-syn and Aβ; however, the causal relationship between these protein aggregates and neuronal cell loss is unclear, especially in PD because patients with PARK2 typically display pure nigral degeneration without LBs, and LB pathology may not be present in patients with dominantly inherited familial PD with LRRK2 and VPS35 mutations (Hayashi et al., 2000; Hasegawa et al., 2009; Bono et al., 2020). In that sense, dissecting the molecular mechanisms responsible for changes in retromer-mediated synaptic neurotransmission and mitochondrial dynamics may help to clarify the pathophysiological cascades of neurodegenerative disorders. Of course, other scenarios that we might not anticipate are also possible. Although stabilization of the retromer complex by pharmacological chaperones can direct disease-causing proteins away from a pathogenic pathway and mitigate neurodegeneration both *in vivo* and *in vitro*, we must continue to decipher the mechanism by which the distinct retromer components and their associated proteins cooperatively function in endosomal sorting and the change of cellular circumstances after machineries are perturbed. Further investigation will uncover the underlying molecular mechanisms of retromer-mediated neurodegeneration and provide a crucial insight into the development of disease-modifying therapy.

## Author Contributions

SY and TH wrote the manuscript. TH provided expertise and edited the manuscript. Both authors contributed to the article and approved the submitted version.

## Conflict of Interest

The authors declare that the research was conducted in the absence of any commercial or financial relationships that could be construed as a potential conflict of interest.

## Publisher’s Note

All claims expressed in this article are solely those of the authors and do not necessarily represent those of their affiliated organizations, or those of the publisher, the editors and the reviewers. Any product that may be evaluated in this article, or claim that may be made by its manufacturer, is not guaranteed or endorsed by the publisher.
